# Green Technology: Bacteria-Based Approach Could Lead to Unsuspected Microbe–Plant–Animal Interactions

**DOI:** 10.3390/microorganisms7020044

**Published:** 2019-02-06

**Authors:** Daniela Bulgari, Matteo Montagna, Emanuela Gobbi, Franco Faoro

**Affiliations:** 1 Department of Agricultural and Environmental Sciences—Production, Landscape, Agroenergy, University of Milan, Italy, via Celoria 2, 20133 Milan, Italy; daniela.bulgari@gmail.com (D.B.); matteo.montagna@unimi.it (M.M.); 2 Piattaforma di Microbiologia Agroalimentare ed Ambientale (Pi.Mi.A.A.), AgroFood Lab, Department of Molecular and Translational Medicine, University of Brescia, 25121 Brescia, Italy; emanuela.gobbi@unibs.it

**Keywords:** plant microbiome, pathogens, trans-kingdom, biostimulants, biocontrol

## Abstract

The recent and massive revival of green strategies to control plant diseases, mainly as a consequence of the Integrated Pest Management (IPM) rules issued in 2009 by the European Community and the increased consumer awareness of organic products, poses new challenges for human health and food security that need to be addressed in the near future. One of the most important green technologies is biocontrol. This approach is based on living organisms and how these biocontrol agents (BCAs) directly or indirectly interact as a community to control plant pathogens and pest. Although most BCAs have been isolated from plant microbiomes, they share some genomic features, virulence factors, and trans-kingdom infection abilities with human pathogenic microorganisms, thus, their potential impact on human health should be addressed. This evidence, in combination with the outbreaks of human infections associated with consumption of raw fruits and vegetables, opens new questions regarding the role of plants in the human pathogen infection cycle. Moreover, whether BCAs could alter the endophytic bacterial community, thereby leading to the development of new potential human pathogens, is still unclear. In this review, all these issues are debated, highlighting that the research on BCAs and their formulation should include these possible long-lasting consequences of their massive spread in the environment.

## 1. Introduction

Biocontrol is defined as the use of living organisms to control pests, also resulting in plant growth promotion [1,2] (Table 1). This approach to pest containment has experienced a revival in the last decade because of the new guidelines for the Common Agricultural Policy (Dir 128/2009) issued in 2009 by the European Community. This directive contains the Integrated Pest Management (IPM) principles (annex III), one cornerstone of which is the promotion of sustainable biological, physical, and other non-chemical methods instead of chemical ones, whenever they provide satisfactory pest control. A greater emphasis on IPM as part of agricultural policy has been giving new input in developing commercial products based on living organisms, with a particular interest in those isolated from rhizosphere, soil, or plant phyllosphere and endosphere.

The first-generation of commercial biocontrol products, mainly based on *Bacillus* and *Pseudomonas* species, were characterized by low efficacy to control plant diseases in open fields [14,15]. The failure of these products was possibly due to the lack of knowledge about (i) biocontrol mechanisms, (ii) plant–microbe interactions, and (iii) selection procedures for active biocontrol strains [14,16]. Biocontrol of plant diseases is a complex process involving not only the biocontrol agent (BCA), the plant, and the pathogen but also the environment, the genetic determinants, and the indigenous microflora (Table 1). Therefore, the effectiveness of BCAs is related to many factors such as their ability to colonize, survive, and proliferate for a considerable time inside and/or on plant tissues in the presence of indigenous microflora and, at the same time, directly or indirectly antagonize phytopathogens [15]. Furthermore, commercial BCAs, registered as such (see ahead), should have some additional properties, i.e., easiness of formulation, the ability to effectively colonize the host and survive in the agricultural environment, and not being pathogenic for non-target organisms [17]. Bacteria can directly antagonize pathogens by competition for root niches or by producing allelochemicals, such as siderophores, antibiotics, biocidal, lytic enzymes, and detoxification enzymes, or by interbacterial antagonism via the type VI secretion system (T6SS) [15,18,19,20]. The secretion system is a particular kind of molecular weapon that delivers antimicrobial peptide in the periplasm or cytoplasm of a recipient bacterium [21,22]. For example, NADase effector family and iron chelator pyoverdine are identified as antimicrobial peptides mediating antagonism via T6SS, respectively, in *Pseudomonas protegens* and *Pseudomonas taiwanensis* [20,23]. Different antibiotic compounds have been isolated from *Pseudomonas*, *Bacillus*, *Paenibacillus*, *Streptomyces*, and *Stenotrophomonas* spp. (among others [24,25,26,27,28]). These compounds are produced by bacteria in a specific metabolic status that is influenced by nutrient availability, pH, temperature, and genetic stability/instability of the bacteria [29]. Furthermore, biotic conditions, plant growth, development, genotype, and presence of competing species can influence antibiotic biosynthesis [18,19,20,21,22,23,24,25,26,27,28,29,30]. BCAs can also protect plants indirectly by inducing the host defense pathways. This phenomenon is called induced systemic resistance (ISR) and confers an enhanced defensive capacity to the plant (reviewed in [31]). The plant response leads to cell wall reinforcement, production of antimicrobial phytoalexins, and synthesis of pathogenesis-related proteins (PR). Interestingly, an enhanced plant capacity to express defense responses occurs only upon challenge inoculation with a pathogen in a mechanism known as ‘priming’ [32]. Thus, in primed plants, defense responses are not activated directly, but are accelerated upon pathogen or insect attack, avoiding fitness costs in the absence of challenge. 

BCAs have been studied for their ability to control or lessen plant pathogens, but little is known about their impact on human health and the environment. As an example, *Bacillus* species antagonize pathogens via the production of secondary metabolites, such as lipopeptides [33,34]. The toxicity of these metabolites has just started to be tested by in vivo assays (i.e., zebrafish model), showing low toxicity to aquatic species in the case of bacillomycin DC isolated from *Bacillus amyloliquefaciens* [35]. Since the regulations of the European Union do not define a specific and exclusive legislative/legal framework for such beneficial microorganisms, they can be registered either as plant protection products or as biofertilizers, phytostimulators, and biopesticides according to national law (Table 1). The registration as biofertilizers, phytostimulators, and biopesticides, instead of BCAs, greatly reduces the set of toxicity tests normally required for plant protection agrochemicals [3,4,5,6]. As emphasized by Yakin and colleagues [3], the lack of a legislative/legal framework opens topical questions, such as: Which is the best category for cultures of living microorganisms? Which standards of proof of efficacy and safety are appropriate to both stimulate the development of these products and safeguard human and environmental health?

## 2. Plant Microbiome: A Fascinating Source of BCAs

With the first results of The Human Genome Project [36], it was immediately clear that humans are composed of a combination of human cells and microorganisms, and that this intimate relationship plays a role in the human physiology and health state [37,38,39]. This idea was translated into the plant kingdom: the Plant Microbiome has been defined not only as a group of microbes associated with a plant but also their ‘crosstalk’ with plant genome and proteome [9,40] (Table 1). Plants teem with microbes associated with the rhizosphere, phyllosphere, endosphere, and with those that adhere to external surfaces. Interestingly, plant microbiomes are structured and form complexes that are interconnected as a network. Inside this system, key taxa have a role in plant fitness, soil fertility, nutrient uptake, plant function, productivity, trait expression (phenotype), environmental plasticity, and health [9,40]. This innovative concept of a plant as a supraorganism is, in some way, responsible for the development of environmentally friendly approaches, such as BCAs, to control plant disease and to increase plant productivity [41]. Among BCAs, the endophytes are certainly the most promising group, since they are microorganisms that colonize internal plant tissues for all, or part, of their lifetime, thus being strictly part of the plant microbiome.

In early works, endophytes were isolated on growth culture media after surface disinfection of different plant tissues. Methods for their isolation have been reviewed extensively [42,43]. Afterwards, the development of cultivation-independent fingerprinting molecular methods based on the 16S rRNA gene allowed a more specific and detailed description of the microbial diversity in complex communities. Nevertheless, our understanding of microbiota complexity has been achieved mainly through the use of ‘next-generation’ and now ‘third-generation’ technologies (among others [8,44,45,46]). These technologies have been used not only to investigate the microbiota composition but also its relationships with the host and the environment [47,48,49,50,51]. Nonetheless, the effect of BCA treatment on endophytic bacterial community composition and activity has been poorly investigated. Hardoim and colleagues [8] have given a comprehensive overview of prokaryotic and eukaryotic endophytes reported in the literature. Despite the extensive amount of sequences analyzed, the major part of endophytes reported belongs to four phyla: Proteobacteria, Actinobacteria, Firmicutes, and Bacteroidetes. Most of the prokaryotic endophytes could be assigned to the Gammaproteobacteria (26%) that are largely represented by a few genera: *Pseudomonas*, *Enterobacter*, *Pantoea*, *Stenotrophomonas*, *Acinetobacter*, and *Serratia*. A similar scenario has also been depicted for eukaryotic endophytes which belong mainly to the Glomeromycota (40%), Ascomycota (31%), Basidiomycota (20%), Zygomycota (0.1%), and unidentified phyla (8%).

Microbiota composition is influenced by different parameters, such as plant genotype and seasonality. Plant genotype, growth stage and physiological status, type of plant tissue, environmental conditions, and agricultural practices also determine endophytic colonization and endosphere community structures (among others [52,53,54,55,56]). Furthermore, metagenomic approaches and comparative genomic analyses allow the identification of intrinsic bacterial traits important for host colonization and for the endophytic lifestyle [8,57,58]. In particular, Hardoim et al. [8] highlighted that endophytic bacteria share genes related to motility and chemotaxis, detoxification, and stress-related enzymes, transporters, and secretion systems. As an example, type IV secretion systems and conjugal DNA–protein transfer secretion systems were detected more prominently among endophytes than among rhizosphere bacteria and phytopathogens. These types of secretion systems are involved in host colonization and conjugation of DNA [59,60].

**Figure 1:**
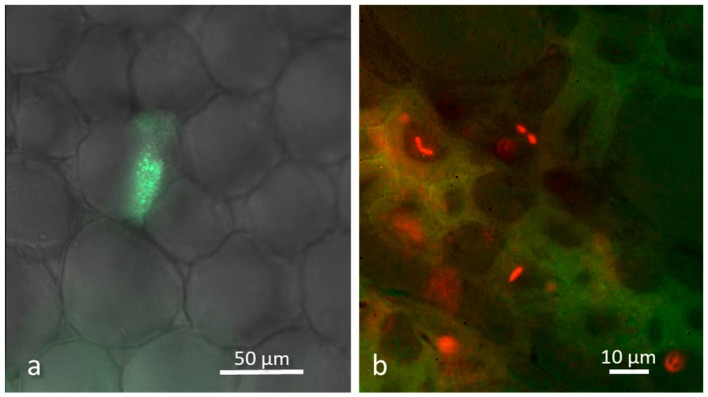
Confocal microscopy images of experimentally inoculated plants showing the ability of endophytic bacteria to inhabit a new host (**a**) *Burkholderia* sp. isolated from grapevine leaf tissues [76] and transformed with green fluorescent protein is able to sustain bacterial cell division in periwinkle parenchyma stem cells. (**b**) *Pantoea agglomerans* isolated from orchids and transformed with red fluorescent protein [77] has been inoculated via root absorption in apple plantlets: bacterial cells are visible in the upper leaves after two weeks from inoculation, demonstrating the ability to stably colonize a different host.

In the last decade, bacteria host colonization process has been extensively reviewed and three main steps were identified: adhesion, penetration, and establishment [8,61,62,63]. Root colonization is strictly linked to root exudation [64]. These exudates are rich in carbohydrates, amino acids, and organic acids that are attractive nutrient sources for bacteria [65]. Microorganisms are chemoattracted by the exudates, allowing them to colonize roots. In turn, the microbiome influences root exudates [66]. By the use of microscopic tools, such as immunomarkers and fluorescence in situ hybridization [67], bacteria have been visualized at an early stage of infection as single cells attached to the root surface, and subsequently as doublets on the rhizodermis, forming a string of bacteria [63,68]. Once inside the plant, endophytic bacteria remain localized in a specific tissue, such as the root cortex, or colonize the plant systematically by transport or active migration through the conducting elements or the apoplast [69,70] (Figure 1). The different mechanisms of distribution might be due to interactions with other bacteria or to the different requirements of each microorganism, allowing them to inhabit different niches, represented by tissues and, more specifically, by the intercellular spaces within each tissue [71]. Furthermore, comparative genome analyses highlighted the presence of genes involved in plant adhesion and penetration, such as hemagglutinin genes, curli fiber genes, and genes related to plant polymer degradation enzymes [72]. Not all bacteria that reach the rhizosphere are competent to become an endophyte. In fact, the key step to becoming a true endophyte is the so-called ‘establishment’, that requires a stable relationship with the host which has to recognize and communicate with the microbiota, and vice versa [73]. It seems that at the beginning, endophytes are recognized as alien organisms, inducing microbe-associated molecular patterns (MAMPs)-triggered immunity (MTI) [74,75]. Subsequently, they are able to secrete effector proteins that suppress plant MTI responses in order to carry on the colonization process [75].

## 3. Human and Animal Pathogens Associated with Plants: Simple Contamination or Survival/Spreading Strategies

Microbiota associated with plants, including bacteria and fungi, have been extensively studied in order to find new microorganisms suitable for plant protection, growth promotion, industrial and medical applications, pollution control, and phytoremediation [78]. Among the numerous studies on this research topic, only a few pointed out that plants can harbor some human and animal pathogens (HAP) [46,62,79,80,81]. For a long time, the scientific community believed that human and plant pathogenic bacteria reside in separate hosts, interacting with them in a specific way as a result of coevolution [79]. However, recent studies have begun to show that many plant pathogens have the ability to colonize other hosts outside of the plant kingdom, including insects, animals, and humans [82,83]. A clear example of cross-kingdom host jumps is *Agrobacterium tumefaciens. A. tumefaciens* was first characterized as the etiological agent of crown gall in rosaceous susceptible genotype [84] and later as a human pathogen [85]. *A. tumefaciens* is able also to transfer DNA not only into plants, but into fungal and human genomes, revealing the ability to infect different hosts belonging to different kingdoms [86,87]. 

The genera *Pantoea* and *Burkholderia* have been recognized as plant-associated bacteria inducing plant diseases, growth promotion, and/or plant protection depending on the agroecosystem [88]. Despite these microbe–plant interactions, some species of *Pantoea* and *Burkholderia* infect humans, causing septicemia, chronic granulomatous disease, melioidosis, arthritis, and urinary infections in immunocompromised humans [79]. The *Burkholderia* genus includes over 60 species which have been found in a variety of ecological niches, ranging from hospitals to humid environments (among others [89,90]). The genus *Burkholderia* has gained considerable importance owing to its potential in biotechnology applications [88]. Due to its pathogenicity in humans and animals, great efforts have been made to unambiguously discriminate plant pathogenic from non-pathogenic strains, including multilocus sequence analyses and comparative genome analyses [90,91,92]. Based on phylogenetic analyses, the scientific community has recently discussed the possibility of dividing this genus into at least two large clusters, including the cluster of plant or animal/human pathogens and the cluster of plant-associated species [89,90]. Interestingly, Estrada-de los Santos and colleagues [93] described an intermediate cluster between the abovementioned groups. This evidence, in association with the ability of bacteria to exchange genes and to evolve rapidly, highlights the need to deepen our knowledge about the plant-associated *Burkholderia* strains and their potential for pathogenicity in animals and humans, and to understand whether gene exchange occurs between the symbiotic and pathogenic *Burkholderia* species. To date, the genetic features characterizing these helpful microorganisms versus the pathogenic ones are not clearly identified and the topic is controversial. It seems that the behavior of the different bacteria is related to gene expression, rather than to the presence or absence of specific genes [94,95].

The plant environment is also a niche for ‘true’ human and animal pathogens (HAPs), such as *Salmonella enterica* serovar Typhimurium and *Escherichia coli* O157:H7, as well as for pathogens that cause diseases in debilitated or immune-compromised humans. Over the past two decades, these so-called opportunistic or facultative human pathogens have had an increasing impact on human health [96,97]. Opportunistic HAPs belonging to different genera, such as *Enterobacter*, *Salmonella*, *Pantoea*, *Serratia*, *Burkholderia*, *Klebsiella*, *Clostridium*, and *Staphylococcus* were found in the rhizosphere and associated with different plant organs [46,61,79,98,99,100]. The occurrence of HAPs in the rhizosphere has been ascribed to several factors, including the nutrient-rich environment, protection from UV radiation, and the availability of water films for dispersal and to prevent desiccation [61,96,101].

The presence of HAPs in plants and outbreaks of foodborne illnesses open new questions about HAP ecology and about the role of plants in the infection cycle [102]. The Enterobacteriaceae family and, especially, the genera *Salmonella* and *Escherichia*, have been extensively studied due to the evidence that they cause major foodborne illnesses. A typical example is verocytotoxigenic *E. coli* (VTEC), which is a foodborne pathogen that can cause serious diseases ranging from hemorrhagic colitis to life-threatening hemolytic uremic syndrome (HUS) and central nervous system damage [103]. Although most cases had been previously associated with contamination of meat, milk products, and eggs, in recent years, fresh fruits and vegetables have been increasingly identified as sources of infection (CDC, Centers of Disease and Control Prevention). Several multistate outbreaks of *E. coli* serotype infections have been reported by both CDC and Food and Drug Administration (FDA) as having arisen from fresh vegetables. Plant contamination by HAPs can occur at different steps from the agricultural environment to the table. HAPs can contaminate vegetables both pre- and post-harvest through irrigation water, farm workers with limited means of proper sanitation, fertilization with slurry, and manure and fecal contamination in the farm by animals [13,104,105,106]. Furthermore, during the post-harvest step, HAPs can contaminate plants during carriage, processing, or packaging [104], and it has also been demonstrated that HAPs can escape post-harvest treatments to control plant pathogens [107,108]. 

Indeed, it has been shown that these so-called contaminations are only a first step in the plant colonization, possibly facilitating the transmission of HAP strains from the field via the food production chain to the consumer with severe impacts on the health of animals and human beings [109]. In this view, plants are recognized as vectors of human pathogens causing phytonoses, a new term for defining the new group of diseases caused by human pathogens, viz., *E. coli* and *S. enterica*, that are transmitted via consumption of fresh produce [12,13] (Table 1). 

The HAP colonization process of a plant is quite similar to plant bacterial colonization, including adhesion, invasion, and establishment [62,110]. The internalization and survival of bacteria in plants represent a food safety threat in crop production, as internalized bacteria cannot be removed by standard sanitation practices, although treatments such as irradiation, ultrasound, and cold plasma can be effective [111,112,113]. It has been demonstrated that *E. coli* O157:H7 can enter in the apoplast of lettuce and spinach from roots and leaves, invade plants, and survive for over 20 days [114,115]. These data, in association with the ability to form biofilm, led to the consideration of these parameters as food risk markers.

## 4. Bacteria Can Overcome Kingdom Barriers

As mentioned above, bacteria host colonization occurs in three main steps: adhesion, penetration, and establishment. Van Baarlen and colleagues [87] highlighted the most important requirements for establishing a pathogenic relationship. These are mainly based on: (i) proximity between microorganism and host, (ii) host ability to act as substrate, (iii) molecular components secreted by bacteria, and (iv) ability to suppress or avoid host immune responses. Despite fundamental differences, the innate immune systems in different eukaryotic kingdoms share a number of common features. These include the structure of molecules involved in microbial recognition; the signaling pathways mediated by mitogen-associated protein kinase; the presence of reactive oxygen species and antimicrobial peptides and proteins [87]. Microbial recognition can occur through so-called microbial or pathogen-associated molecular patterns (MAMPs/PAMPs), which include different types of molecules, such as the lipopolysaccharides of Gram-negative bacteria and the peptidoglycans of Gram-positive bacteria, as well as bacterial flagellin, microbial DNA, and fungal cell wall constituents [31,116]. Moreover, plant–pathogen recognition is mediated by effector molecules injected or secreted into the host cells by secretion systems. Some systems secrete a variety of substrates, while others are only found in a small number of bacterial species and/or are specific to one or few types of proteins [117]. In plants and animals, MAMPs and effectors are recognized by both cell surface receptors [118] and intracellular receptors of the NLR (nucleotide-binding domain (NBD) and leucine-rich repeat (LRR)) superfamily [119,120,121]. Microbial recognition mechanisms by plants and animals and their NLR architecture overlap, and it is now possible to discern important key trans-kingdom principles of NLR-dependent immune function [87]. For example, *S. enterica* induces MAMP-triggered immunity in *Arabidopsis thaliana* via flagellin FLS2 recognition [122]. Plants and animals share other similarities in host defense signaling after pathogen perception [123], and one class of antimicrobial peptides comprising the defensins is found to be conserved across kingdoms [124]. 

Finally, cross-kingdom bacteria should be able to survive and live in the future hosts. Survival upon entering a new host is associated with an innate ability to change the metabolic activity or adapting to (and taking advantage of) host metabolism. *S. enterica, Pseudomonas aeruginosa, Burkholderia cepacia,* and *E. coli* are the most deeply investigated cross-kingdom pathogens [105]. *S. enterica* is mainly transmitted via water, from feces to environment and then to crop [105]. Once it reaches plant tissues, it can persist for significant periods of time as it is able to colonize the mesophyll via stomata openings, wounds, and hydathodes, as shown in arugula and tomato plants [98,100,125,126,127]. *S. enterica* and other enterobacteria (e.g., *E. coli)* are able to reprogram the host architecture, suppressing the host immune system via the injection of a cocktail of effector proteins (among others [128,129,130]). The effector proteins and secretion systems of enterobacteria are known, and their function in human and mouse has been described in depth (reviewed in [131]). Interestingly, it has been shown that these proteins are able to suppress plant defense also in tobacco plants and *Arabidopsis thaliana* [132,133]. In particular, *Salmonella* T3SS effectors (Type III secretion system) are essential for both animal pathogenicity and the plant colonization process [132,133,134,135]. Recently, Neumann and colleagues [129] demonstrated that the phosphothreonine lyase SpvC attenuates the induction of immunity-related genes, allowing bacteria proliferation in *Arabidopsis thaliana.* This evidence strongly confirms the idea that plants could be reservoirs or vectors of human pathogens.

## 5. Possible Unfavorable Consequences of the Massive Use of Bacteria as Biocontrol or Biofertilizer Agents

The concept of the ‘pathobiome’ has been introduced to define the role of the microbiome in causing pathogenesis, replacing the dogma ‘one microbe—one disease’ [11,78]. This term was coined based on metagenomic data showing the complexity of the microbial communities associated with the ecological niches inhabited by pathogens. Disease development can be then influenced by variations in host-associated microbial populations, signaling within bacterial communities, and the immune state of the host [136,137,138,139]. In other words, pathogenicity is an outcome of host–microbe interactions, thus inextricably linked to the host and microbe characteristics [140]. As a consequence, the separation between beneficial and detrimental microorganisms is not that clear-cut, due to the fact that horizontal gene transfers (HGTs) may confer virulence traits to harmless bacteria [141,142]. 

As discussed in the previous paragraphs, most BCAs are close relatives of HAPs. Moreover, comparative genome analyses have shown that endophytic bacteria, often recommended as BCAs, share genome structures and distributions of virulence genes with pathogenic bacteria [92,94]. This evidence suggests the ability of these bacteria to change their lifestyle according to the ecological niche. For example, the genus *Paenibacillus,* a promising genus for biocontrol, exhibits extensive environmental adaptability and can populate various ecological niches [28]. Comparative genome analyses of *Paenibacillus* spp. revealed that this capacity is related to a highly diverse gene repertoire and to the HGT trend [143]. 

**Figure 2:**
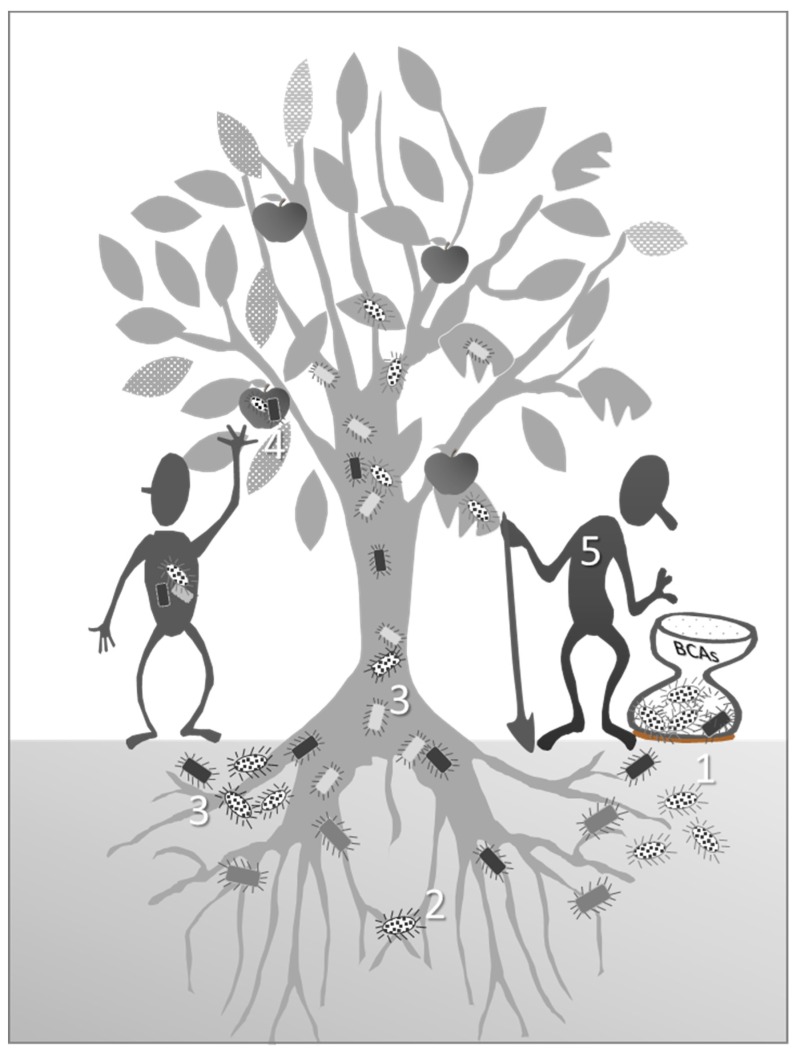
Schematic representation of a potential scenario following plant disease/growth biocontrol: (1) Biocontrol agents (BCAs, in dotted white), differently formulated, are spread into the soil; (2) they interact with soil and plant microbiomes (in gray and light gray, respectively) improving plant health and fitness; (3) BCAs can also interact with human pathogens (in black) harbored by plants, possibly leading to horizontal gene transfer (i.e., resistance to antibiotics); (4) these bacteria could migrate through the plant up to the edible parts, whose consumption may lead to severe diseases, such as septicemia and urinary infection; (5) finally, BCAs potentially pathogens for humans and animals (in black) can contaminate the farm workers or the post-harvest process, entering into the food chain.

From this perspective, the massive use of bacterial BCAs to control pathogens or as biofertilizer could affect disease development and increase the possibility of new pathogen emergence by altering the host-associated microbiome and/or the host immune system. Moreover, the potential direct pathogenicity of putative BCAs to human health has been often underinvestigated, even if some of them (e.g., *Burkholderia* spp., *Paenibacillus* spp., and *Pseudomonas* spp.) have been reported as opportunistic human or animal pathogens, or are known to produce secondary metabolites potentially toxic for humans and animals (Figure 2). For example, *Paenibacillus polymyxa* is reported in the literature as a prominent biofertilizer or biocontrol agent [144], even if this species produces cyclic lipodepsipeptide fusaricidins that are toxic to mitochondria and induce apoptosis in mammalian cells [145]. 

This evidence, in combination with the outbreaks of human infections associated with the consumption of raw fruits and vegetables, opens new questions regarding the role of plants in the human pathogen infection cycle, or if a biological control could alter the endophytic bacterial community, leading to the generation of new potential human pathogens.

## 6. Conclusions

Bacteria are essential components of human, animal, and plant health, and important sources of new molecules suitable for industrial, medicine, and agricultural applications. Nevertheless, some of the endophytic bacteria and fungi used in biocontrol or biofertilization strategies are indeed true or opportunistic human and animal pathogens, or carry human virulence factors in their genome. Thus, their massive spreading in the soil and in the environment, with scarce or no knowledge of their interactions with plants and with the phyllosphere and rhizosphere microbiomes, may lead to unpredictable long-term consequences. Although biocontrol is a promising approach to controlling plant pathogens, it is important to re-think the assumption ‘isolation from plant is safe’, taking into account the possible direct or indirect effects on human health and the environment, which have so far been unappreciated. Nowadays, the omics technologies can hopefully contribute to deeply understanding the new bacteria–plant–animal interactions and, thus, their impact on agroecosystems and human health. Omics technologies can contribute to increase this knowledge constituting the starting point for testing predictive hypotheses on microbial pathogenicity in model systems. 

## Figures and Tables

**Table 1:** Terminology and definitions related to biocontrol field.

Term	Definition	Reference
***Biocontrol***	The use of living organisms to control plant pathogens and pests resulting in plant growth promotion	[2]
***Biostimulants***	Formulated products with novel, or emergent properties due the complex of constituents, that improve plant productivity not as a sole consequence of the presence of known essential plant nutrients, plant growth regulators, or plant protective compounds	[3]
***Biopesticide***	Biological pesticides are derived from natural materials including plants, animals and microbe, and some minerals	[4]
***Biofertilizer***	A biofertilizer is any bacterial or fungal inoculant applied to plants with the aim of increasing the availability of nutrients and their utilization by plants, regardless of the nutrient content of the inoculant itself. Biofertilizers may also be defined as microbial biostimulants improving plant nutrition efficiency.	[5]
***Phytostimulator***	Microorganism with the ability to produce or change the concentration of growth regulators, such as indole acetic acid, gibberellic acid, cytokinins, and ethylene	[6]
***Pathogen***	A microbe that can cause physiological and structural damages in a host	[7]
***Biocontrol agents***	Living organisms that show the ability to directly or indirectly antagonize plant pathogens and pests	here defined
***Endophytic bacteria***	Bacteria that habit for all or part of their lifetime, in the internal part of a plant	[8]
***Plant microbiome***	A community of microbes associated with a plant and their crosstalk with the plant genome and proteome	[9]
***Pathobiome***	The complex interactions of pathogenic microbes which may influence or drive disease processes and their relationship to the ‘normal’ microbiome of the organism in question	[10,11]
***Phytonoses***	New group of diseases caused by human pathogens that are transmitted via consumption of fresh produce	[12,13]
